# Distinct Patterns of IFITM-Mediated Restriction of Filoviruses, SARS Coronavirus, and Influenza A Virus

**DOI:** 10.1371/journal.ppat.1001258

**Published:** 2011-01-06

**Authors:** I-Chueh Huang, Charles C. Bailey, Jessica L. Weyer, Sheli R. Radoshitzky, Michelle M. Becker, Jessica J. Chiang, Abraham L. Brass, Asim A. Ahmed, Xiaoli Chi, Lian Dong, Lindsay E. Longobardi, Dutch Boltz, Jens H. Kuhn, Stephen J. Elledge, Sina Bavari, Mark R. Denison, Hyeryun Choe, Michael Farzan

**Affiliations:** 1 Department of Microbiology and Molecular Genetics, Harvard Medical School, New England Primate Research Center, Southborough, Massachusetts, United States of America; 2 US Army Medical Research Institute of Infectious Disease, National Interagency Biodefense Campus, Frederick, Maryland, United States of America; 3 Departments of Pediatrics and Microbiology and Immunology and Elizabeth B. Lamb Center for Pediatric Research, Vanderbilt University Medical Center, Nashville, Tennessee, United States of America; 4 Ragon Institute of Massachusetts General Hospital, Massachusetts Institute of Technology, and Harvard Medical School, Charlestown, Massachusetts, United States of America; 5 Department of Pediatrics, Harvard Medical School, Children's Hospital, Boston, Massachusetts, United States of America; 6 Integrated Research Facility at Fort Detrick, National Institute of Allergy and Infectious Diseases, National Institutes of Health, National Interagency Biodefense Campus, Frederick, Maryland, United States of America; 7 Tunnell Consulting Inc., King of Prussia, Pennsylvania, United States of America; 8 Department of Genetics, Brigham and Women's Hospital, Howard Hughes Medical Institute, Harvard Medical School, Boston, Massachusetts, United States of America; University of North Carolina at Chapel Hill, United States of America

## Abstract

Interferon-inducible transmembrane proteins 1, 2, and 3 (IFITM1, 2, and 3) are recently identified viral restriction factors that inhibit infection mediated by the influenza A virus (IAV) hemagglutinin (HA) protein. Here we show that IFITM proteins restricted infection mediated by the entry glycoproteins (GP_1,2_) of Marburg and Ebola filoviruses (MARV, EBOV). Consistent with these observations, interferon-β specifically restricted filovirus and IAV entry processes. IFITM proteins also inhibited replication of infectious MARV and EBOV. We observed distinct patterns of IFITM-mediated restriction: compared with IAV, the entry processes of MARV and EBOV were less restricted by IFITM3, but more restricted by IFITM1. Moreover, murine Ifitm5 and 6 did not restrict IAV, but efficiently inhibited filovirus entry. We further demonstrate that replication of infectious SARS coronavirus (SARS-CoV) and entry mediated by the SARS-CoV spike (S) protein are restricted by IFITM proteins. The profile of IFITM-mediated restriction of SARS-CoV was more similar to that of filoviruses than to IAV. Trypsin treatment of receptor-associated SARS-CoV pseudovirions, which bypasses their dependence on lysosomal cathepsin L, also bypassed IFITM-mediated restriction. However, IFITM proteins did not reduce cellular cathepsin activity or limit access of virions to acidic intracellular compartments. Our data indicate that IFITM-mediated restriction is localized to a late stage in the endocytic pathway. They further show that IFITM proteins differentially restrict the entry of a broad range of enveloped viruses, and modulate cellular tropism independently of viral receptor expression.

## Author Summary

Cells express restriction factors, proteins whose primary activity is to inhibit viral replication. We have recently described a family of restriction factors, interferon-inducible transmembrane (IFITM) proteins, that interfere with replication of influenza A virus. The IFITM proteins uniquely inhibit replication early in the viral life-cycle, before the virus can successfully enter the cell cytoplasm. Here we show that the entry processes of several highly pathogenic viruses – Marburg virus, Ebola virus, and SARS coronavirus – are similarly disrupted by IFITM proteins. We compared IFITM-mediated restriction of these viruses with influenza A virus, and discovered that individual IFITM proteins are specialized for restriction. For example, we describe two mouse IFITM proteins that efficiently restrict entry of Marburg and Ebola viruses, but which do not inhibit influenza A virus. We further show that we can circumvent IFITM-mediated restriction by inducing a virus to enter a cell at or near the plasma membrane. This observation indicates that restriction is not a global property of the cell, but rather is localized to late endosomal and lysosomal compartments, the usual entry sites of IFITM-restricted viruses. This study therefore enhances our understanding of how the innate immune system controls influenza A virus and other pathogenic viruses.

## Introduction

The interferon-inducible transmembrane (IFITM) proteins are a family of viral restriction factors that play critical roles in the interferon-mediated control of influenza A virus (IAV) [1]. These proteins inhibit both IAV replication and infection by hemagglutinin (HA)-pseudotyped retroviruses, indicating that they target the IAV entry process. IFITM proteins also restrict an early step in the lifecycle of several flaviviruses, including dengue and West Nile viruses. In contrast, they do not inhibit replication of murine leukemia virus (MLV), or the entry processes of amphotropic MLV, Machupo virus (MACV), Lassa virus (LASV), or lymphocytic choriomeningitis virus (LCMV). Although IFITM proteins are induced by type I and II interferons, most cells and cell lines express a basal level of one or more of these proteins [2]. IFITM1, 2, and 3 are expressed in a wide range of tissues, whereas IFITM5 expression appears to be limited to bone [3]. Mice have orthologs for IFITM1, 2, 3, and 5, as well as two additional IFITM genes, *Ifitm6* and *Ifitm7*. *IFITM4P* is a pseudogene in both species [4]. Two IFITM proteins have been identified in chickens, orthologs of human IFITM1 and IFITM5. The IFITM proteins are small (∼130 amino acids), with two transmembrane domains separated by a highly conserved cytoplasmic domain. Both amino- and carboxy- domains are luminal [5].

Enveloped viruses usually express surface proteins that mediate attachment of virions to a cellular receptor. Following receptor engagement, these entry proteins undergo conformational changes that ultimately promote mixing of viral and cellular membrane lipids, formation of a fusion pore, and transfer of the viral genome to the cell cytoplasm [6]. The conformational changes of some entry proteins, for example IAV HA or flavivirus E proteins, are induced by low pH [7], [8]. Fusion mediated by these proteins requires access to an acidic cellular compartment such as a late endosome or lysosome. In contrast, other entry proteins such as the envelope proteins of HIV-1 and MLV do not require access to an acidic compartment, and these viruses are presumed to fuse at the plasma membrane or in an early endocytic vesicle [9]. Finally, some entry proteins, such as the severe acute respiratory syndrome coronavirus (SARS-CoV) spike (S) protein or the filovirus glycoproteins (GP_1,2_), indirectly require low pH to activate target-cell proteases necessary for viral entry [10], [11], [12], [13]. Proteolytic cleavage of these entry proteins by lysosomal cathepsin L (SARS-CoV) or cathepsins B and L (Ebola virus; EBOV) promotes the final steps of viral fusion [14], [15]. In the case of SARS-CoV, the requirement for cathepsin L can be circumvented by treatment of receptor-bound virions with trypsin or related proteases [16]. This observation has been used to suggest alternative pathways for SARS-CoV infection, depending on whether cathepsin L or an extracellular protease activates the receptor-associated S protein.

Here we demonstrate that, like IAV, the entry processes of Marburg virus (MARV), EBOV, and SARS-CoV are specifically restricted by IFITM proteins. We observed that various human, mouse, and chicken IFITM orthologs differentially restricted IAV and filoviruses. We further showed that restriction of SARS-CoV was circumvented by trypsin treatment of cell-associated virions. Our data identify variation in the properties of individual IFITM proteins and localize their restriction activity to a late stage in the endocytic pathway. They further suggest that these proteins restrict a broad range of enveloped viruses, and can modulate cellular tropism independently of viral receptor expression.

## Results

### MARV and EBOV GP_1,2_-mediated entry is restricted by IFITM1, 2, and 3

**Figure 1:**
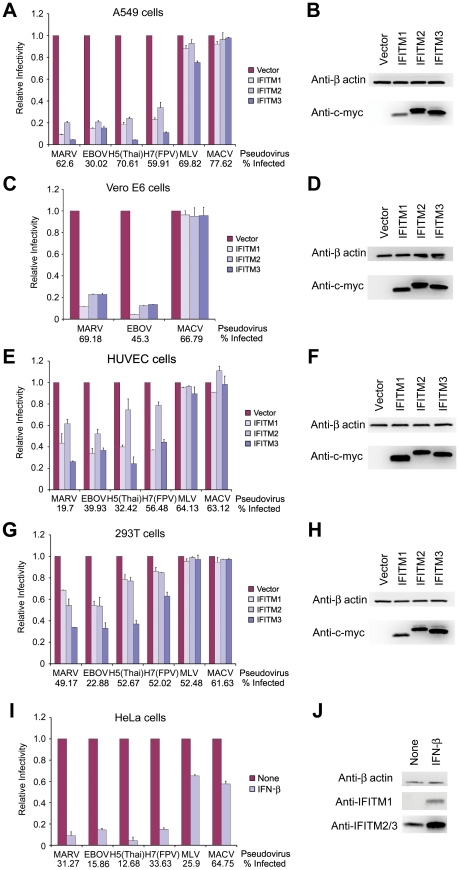
MARV and EBOV GP_1,2_-mediated entry is restricted by IFITM1, 2, and 3. (**A**) A549, (**C**) Vero E6, (**E**) HUVEC, or (**G**) 293T cells transduced to express the indicated c-myc-tagged IFITM proteins or with vector alone were infected with MLV-GFP pseudotyped with the entry proteins of EBOV, MARV, IAV, MLV, or MACV, as indicated. Two days later, pseudovirus infection was determined by flow cytometry. Relative infectivity represents the percentage of GFP-positive cells, normalized to that of cells transduced with vector alone. Numbers underneath figures indicate percentage of infected cells in vector-transduced cells. Differences in pseudovirus entry between vector alone and IFITM expressing cells are significant (*P*<0.05) for all MARV, EBOV, IAV pseudoviruses excepting H7(FPV) in 293T cells expressing IFITM1 (*P*<0.1). IFITM protein expression in (**B**) A549, (**D**) Vero E6, (**F**) HUVEC, or (**H**) 293T cells was measured by western blot with an anti-c-myc antibody (9E10), using aliquots of the same cells assayed in (A), (C), (E), and (G), respectively. β-actin was included as a loading control. (**I**) Experiment similar to (A) except that HeLa cells were treated with 5000 U/ml IFN-β or maintained in growth medium for 48 hours before infection with the indicated pseudoviruses. Differences in pseudovirus entry between MLV or MACV and MARV, EBOV, or IAV are statistically significant (*P*<0.05), as are all differences in pseudovirus entry between IFN-β-treated and untreated cells. (**J**) In parallel, aliquots of the same cells assayed in (I) were used to determine the expression of IFITM proteins. IFITM proteins were analyzed by western blot and probed with the indicated anti-IFITM1 or anti-IFITM2/3 antibody. Each panel of the figure represents at least three experiments with similar results.

The IFITM proteins restrict IAV HA-mediated entry, and an early stage in the replication of several flaviviruses [1]. The entry process of each of these viruses is pH dependent. We sought to determine if additional viruses that require access to late endocytic or lysosomal compartments were similarly restricted by these proteins. The entry processes of MARV and EBOV require activation by one or more cathepsins, whose activity is localized to lysosomes [10], [12], [13], [17]. Both EBOV and MARV require cathepsin L activity to infect cells, whereas EBOV also requires cathepsin B activity [10], [12], [13]. Accordingly, we incubated MLV-based pseudoviruses bearing the entry proteins of MARV, EBOV, H5 or H7 IAV, MLV, or MACV with A549 lung epithelial cells transduced to express human IFITM1, 2 or 3, or with vector alone [18], [19], [20]. As we have previously reported [1], infection mediated by the entry proteins of MLV or MACV was unaffected by the presence of IFITM proteins (Fig. 1A), despite their efficient expression (Fig. 1B). In contrast, as reported, IAV HA-mediated entry was markedly inhibited by the same proteins. Similarly, we observed that entry mediated by MARV and EBOV GP_1,2_ proteins was inhibited by IFITM1, 2 and 3 (Figs. 1A and S1A). Comparable results were obtained in Vero E6 cells, an African green monkey kidney epithelial cell line and the standard cell line for laboratory propagation of these viruses (Figs. 1C and D), and for human umbilical vein endothelial cells (HUVEC), a primary cell target of filovirus infection (Figs. 1E and F). We have repeatedly observed that IFITM-mediated restriction is less efficient in human embryonic kidney 293T cells. Consistent with this observation, both IAV HA- and filovirus GP_1,2_-mediated entry were only partially suppressed in these cells, whereas control pseudoviruses remained unaffected (Figs. 1G and H). Our data indicate that IFITM1, 2, and 3 each restrict MARV and EBOV GP_1,2_-mediated entry, and identify cell-type differences in the efficiency of restriction. We speculate that these differences arise from expression differences of necessary restriction cofactors or proteins that interfere with IFITM protein activity.

### Type 1 interferon inhibits filovirus GP_1,2_-mediated entry

Expression of IFITM proteins is potently induced by type I or type II interferons (Fig. S1B) [2]. To determine whether the entry processes of MARV and EBOV are similarly regulated by type I IFN, we incubated HeLa cells with IFN-β and then infected cells with pseudoviruses bearing MARV, EBOV, IAV, MACV, or MLV entry proteins (Fig. 1I). As has been previously reported, IFN-β treatment upregulated IFITM proteins (Fig. 1J). Consistent with this higher expression of IFITM proteins, entry mediated by MARV and EBOV GP_1,2_ or IAV HA was markedly inhibited by IFN-β (Fig. 1I). In contrast, infection by MLV and MACV pseudoviruses was only modestly suppressed, an effect possibly due to post-entry inhibition of GFP expression in treated cells. We conclude that that the entry processes of MARV, EBOV, and IAV are inhibited by type I IFN.

### Replication of infectious MARV and EBOV is restricted by IFITM1, 2, and 3

**Figure 2:**
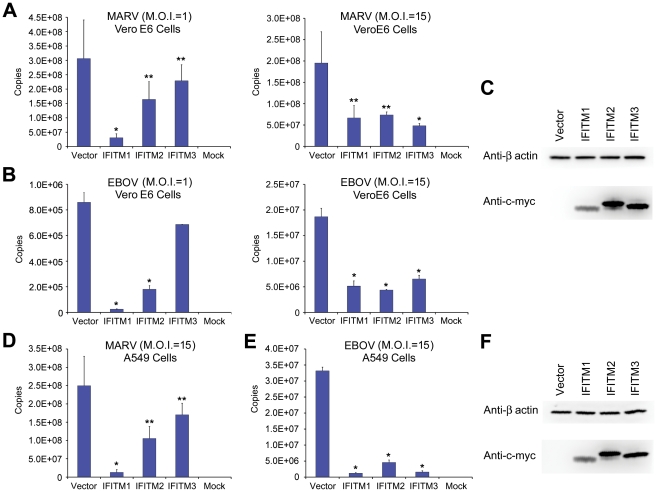
Replication of infectious MARV and EBOV is restricted by IFITM1, 2, and 3. Vero E6 cells transduced to express the indicated c-myc-tagged IFITM proteins or with vector alone were incubated with infectious (**A**) MARV or (**B**) EBOV at indicated MOIs for 1 hour and then maintained in growth medium. After 72 hours, culture supernatant was harvested and viral titer was assayed using quantitative RT-PCR. A single asterisk indicates a significant difference with controls cells (*P*<0.05); double asterisks indicate *P*<0.1. (**C**) IFITM protein expression in Vero E6 cells was assayed by western blot using anti-c-myc antibody. (**D**) and (**E**) are experiments similar to those in (A) and (B), except A549 cells transduced to express the indicated c-myc-tagged IFITM proteins or with vector alone were used. (**F**) IFITM protein expression in A549 cells was assayed by western blot using anti-c-myc antibody. Each panel represents two experiments with similar results.

To determine whether IFITM proteins restrict infectious filoviruses, Vero E6 cells transduced to express IFITM1, 2 or 3, or with vector alone were incubated with infectious MARV or EBOV at MOIs of 1 or 15. As was observed with pseudoviruses, replication of both infectious filoviruses was restricted by expression of IFITM proteins (Figs. 2A–C and S1C), most consistently by IFITM1. Similar results were obtained in A549 cells (Figs. 2D–F). Thus expression of IFITM1, 2, or 3 suppresses replication of infectious MARV and EBOV. Collectively with Fig. 1, these data suggest that suppression of filovirus replication by IFITM proteins and by type I interferons is due in part to inhibition of entry.

### SARS-CoV S protein mediated entry is restricted by IFITM1, 2, and 3

**Figure 3:**
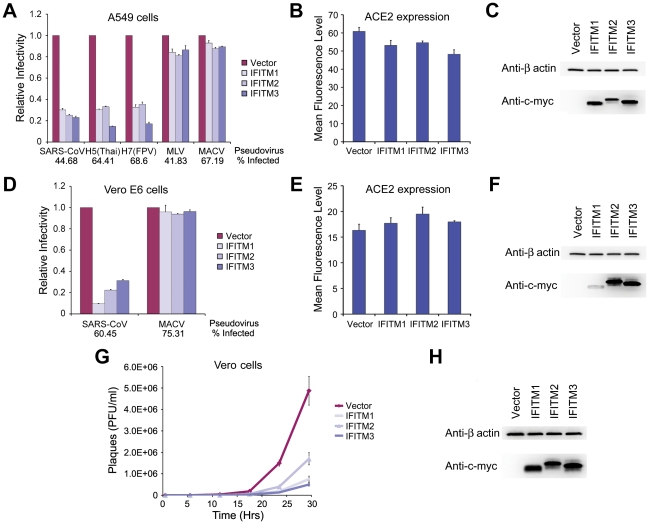
SARS-CoV S infection is restricted by IFITM1, 2, and 3. (**A**) A549 or (**D**) Vero E6 cells transduced to express ACE2 were subsequently transduced to express the indicated c-myc-tagged IFITM proteins or with vector alone. Two days later, cells were infected with indicated pseudoviruses. Pseudovirus infection was determined by flow cytometry, and normalized to that of cells transduced with vector alone. Differences in pseudovirus entry between vector alone and IFITM expressing cells are significant (*P*<0.05) for all SARS-CoV and IAV pseudoviruses. In parallel, cell-surface expression of ACE2 was assayed using aliquots of the same (**B**) A549 or (**E**) Vero E6 cells analyzed in (A) and (D), respectively. Cells were labeled with Alexa-488 conjugated S protein RBD of SARS-CoV and analyzed by flow cytometry. ACE2 expression is shown as mean fluorescence intensity. Expression of c-myc-tagged IFITM proteins was assayed by western blot using aliquots of the same (**C**) A549 or (**F**) Vero E6 cells analyzed in (A) and (D), respectively. Each panel represents at least three experiments with similar results. (**G**) Vero cells transduced to express indicated c-myc-tagged IFITM proteins or with vector alone were incubated in duplicate with infectious SARS-CoV at a MOI of 0.1 for 1 hour. Supernatants were harvested 1, 6, 12, 18, 24, or 30 hours later and viral titers were measured by plaque assay. (**H**) Expression of c-myc-tagged IFITM proteins was assayed by western blot using aliquots of cells analyzed in (G).

As is the case for both MARV and EBOV, SARS-CoV requires cathepsin L activity to enter cells [10], [11]. We therefore investigated whether IFITM proteins could restrict SARS-CoV S protein-mediated entry. To do so, we first introduced the SARS-CoV receptor, angiotensin-converting enzyme 2 (ACE2), into A549 cells before transducing them to express IFITM1, 2, or 3, or with vector alone [21]. Entry mediated by the SARS-CoV S protein, like IAV HA-mediated entry, was restricted by each IFITM protein, whereas MLV and MACV pseudoviruses were unaffected (Fig. 3A). IFITM expression did not substantially interfere with cell-surface expression of ACE2, indicating that suppression is not due to receptor down-regulation (Fig. 3B and C). Similar results were obtained in Vero E6 cells (Figs. 3D, E, and F). Consistent with SARS-CoV pseudoviruses, infectious SARS-CoV replicated in control Vero cells markedly more efficiently than in cells expressing IFITM1, 2, or 3 (Figs. 3G and H). We conclude that, like EBOV and MARV, SARS-CoV entry can be restricted by each IFITM.

### Depletion of IFITM proteins differentially enhances infection mediated by MARV, SARS-CoV, and IAV entry proteins

**Figure 4:**
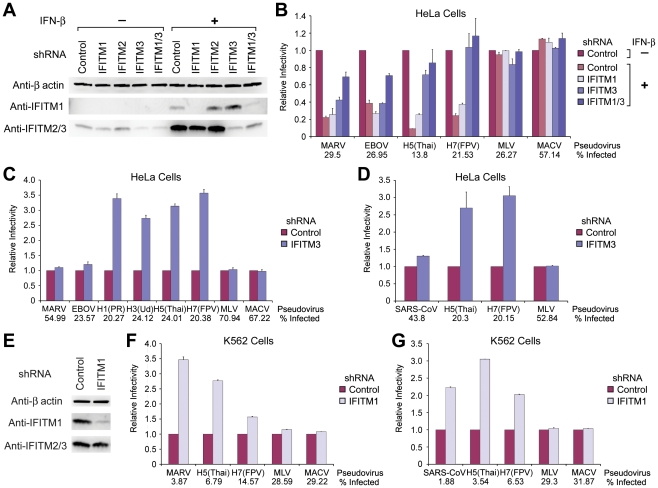
Depletion of IFITM proteins differentially enhances infection mediated by MARV, EBOV, SARS-CoV, and IAV entry proteins. (**A**) HeLa cells were transduced to express control shRNA or shRNA targeting IFITM1, 2, 3, or IFITM1 and 3 (IFTIM1/3), and selected by puromycin. HeLa cells were then treated with 1000 U/ml IFN-β or with medium alone for 48 hours. Expression of IFITM proteins in control or IFITM depleted HeLa cells was assayed by western blot using the indicated anti-IFITM1 or anti-IFITM2/3 antibody. β-actin was included as a loading control. (**B**) Aliquots of the cells used in (A) were infected with MLV-GFP pseudotyped with the indicated entry proteins. Pseudovirus infection was measured by flow cytometry, and normalized to infection of cells expressing control shRNA in the absence of IFN-β. Differences in pseudovirus entry between IFN-β-treated and untreated cells for MARV, EBOV, or IAV are significant (*P*<0.05), except for IAV entry into HeLa cells expressing IFITM3 or IFITM1/3 shRNA. (**C**) Experiment similar to (B) except HeLa cells stably expressing indicated shRNA were assayed in the absence of IFN-β. (**D**) HeLa cells expressing indicated shRNA were transduced to express ACE2 and infected with indicated pseudoviruses. Differences in pseudovirus entry between cells expressing control and IFITM3 shRNA in (C) and (D) are significant (*P*<0.05) for IAV pseudoviruses only. (**E**) Experiment similar to (A) except that K562 cells were transduced to express control shRNA or shRNA targeting IFITM1, and selected by puromycin. (**F**) Experiment similar to that in (B) except that infectivity of the indicated pseudoviruses was measured in K562 cells stably expressing the indicated shRNA. (**G**) Experiment similar to (D) except that infectivity of the indicated pseudoviruses was measured in ACE2- and shRNA-expressing K562 cells. Differences in pseudovirus entry between cells expressing control and IFITM1 shRNA in (F) and (G) were significant (*P*<0.05) for MARV, SARS-CoV, and IAV pseudoviruses. Each panel of the figure represents at least two experiments with similar results.

Throughout our studies we observed a modest trend in cells over-expressing IFITM proteins in which IFITM3 more efficiently restricted IAV [1], whereas no similar pattern was observed with MARV, EBOV, or SARS-CoV. To further explore differences HeLa cells were transduced to express shRNA targeting IFITM1, 2, 3 or shRNA targeting both IFITM1 and 3 expression. Cells were maintained in the presence or absence of IFN-β and incubated with MARV, EBOV, IAV, MLV or MACV pseudoviruses (Figs. 4A and B). As expected, IFN-β efficiently inhibited entry mediated by MARV and EBOV GP_1,2_ and IAV HA proteins. Depletion of IFITM3 fully restored IAV HA-mediated infection, an effect only modestly enhanced through suppression of IFITM1 expression. In contrast, entry mediated by MARV and EBOV GP_1,2_ was not restored in cells expressing shRNA targeting IFITM1 or IFITM3 expression. However, when both IFITM1 and IFITM3 were depleted, filovirus GP_1,2_-mediated entry was partially restored. These results suggest that IAV entry is more sensitive to IFITM3-mediated restriction whereas IFITM1 plays a more important role in suppressing filovirus entry processes.

We further examined these differing profiles of IFITM restriction in cells endogenously expressing IFITM proteins. HeLa cells express a detectable basal level of IFITM3, but not IFITM1 (Fig. 4A). shRNA targeting IFITM3 markedly enhanced entry mediated by all four IAV HA proteins assayed, but did not substantially increase infection by MARV, EBOV, or control pseudoviruses (Fig. 4C and Fig. S2A). Similarly IFITM-targeting shRNA did not affect entry of SARS-CoV pseudovirus into ACE2-expressing HeLa cells, whereas IAV infection was again markedly enhanced by IFITM3 shRNA (Fig. 4D). ACE2 expression was comparable in control and IFITM3-depleted cells (Fig. S2B). To explore the role of endogenous IFITM1, we used human myelogenous leukemia K562 cells, which express relatively high levels of this IFITM protein (Fig. 4E). Depletion of IFITM1 markedly enhanced entry of MARV and, to a lesser extent, IAV pseudoviruses (Fig. 4F). Similarly, shRNA targeting IFITM1 did not alter ACE2 expression, but enhanced SARS-CoV S protein-mediated entry (Figs. 4G and S2C). Thus, cells can express different basal levels of each IFITM protein, and these levels differentially alter their susceptibility to IAV and cathepsin-dependent viruses.

### Murine and chicken IFITM orthologs differentially restrict infection mediated by MARV, EBOV, and IAV entry proteins

**Figure 5:**
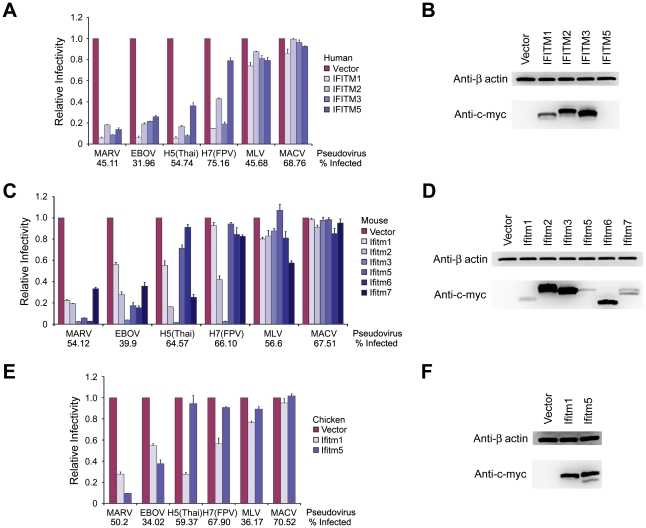
Murine and chicken IFITM orthologs differentially restrict infection mediated by MARV, EBOV, and IAV entry proteins. A549 cells were transduced to express the indicated c-myc-tagged (**A**) human, (**C**) mouse, or (**E**) chicken IFITM orthologs. Two days later, cells were infected with MLV-GFP pseudotyped with the indicated viral entry glycoproteins. Pseudovirus infection was measured by flow cytometry, and normalized to that of cells transduced with vector alone. Expression of (**B**) human, (**D**) mouse, or (**F**) chicken IFITM protein orthologs in A549 cells assayed in (A), (C), and (E), respectively was measured by western blot using the anti-c-myc antibody (9E10). All differences in pseudovirus entry between control and IFITM-expressing cells are significant (*P*<0.05) except for H5(Thai) entry into murine Ifitm6- and chicken Ifitm5-expressing cells, and for H7(FPV) entry into murine Ifitm1- and Ifitm5-expressing cells. Each panel of the figure represents at least two experiments with similar results.

To further explore the extent to which IFITM proteins differentially restrict IAV and filoviruses, A549 cells were transduced to express all known human (Figs. 5A and B), mouse (Figs. 5C and D), or chicken (Figs. 5E and F) IFITM orthologs. Cells were then incubated with pseudoviruses bearing MARV, EBOV, IAV, MLV, or MACV entry proteins. Nearly every IFITM ortholog restricted MARV and EBOV GP_1,2_-mediated entry, despite variation in the expression of these orthologs. Note that human IFITM5 expression, undetectable by western blotting, was observed by immunofluorescent staining (Fig. S3D). In contrast, mouse and chicken IFITM5 and mouse IFITM6 did not efficiently restrict IAV HA-mediated entry. (An alignment of IFITM orthologs is shown in Fig. S3). Unlike the modest differences between human IFITM1 and IFITM3, these differences were sufficient to be observed in over-expression assays. We conclude that IFITM genes have overlapping but distinct effects on the entry of IAV and filoviruses.

### Trypsin treatment bypasses IFITM restriction of entry mediated by SARS-CoV S protein

**Figure 6:**
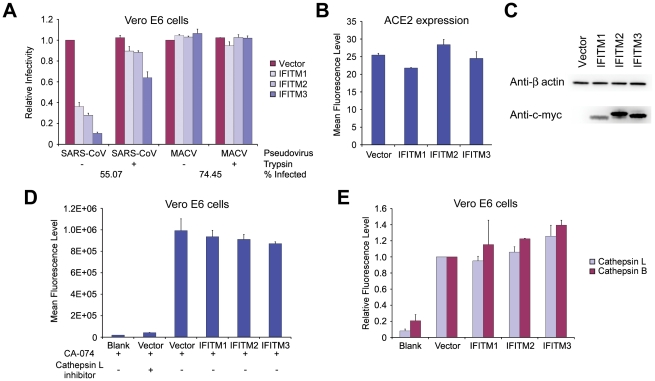
Trypsin treatment bypasses IFITM restriction of entry mediated by SARS-CoV S protein. (**A**) Vero E6 cells transduced to express the indicated c-myc-tagged IFITM proteins or with vector alone were subsequently transduced to express ACE2. Two days later, cells were spin-inoculated at 4°C with indicated pseudoviruses and then treated with 5 µg/ml trypsin or phosphate-buffered saline (PBS) at 37°C for 13 minutes. Infected cells were maintained in growth medium and pseudovirus infection was determined by flow cytometry two days later. Relative infectivity was shown as the percentage of GFP-positive cells, normalized to that of cells transduced with vector alone and treated with PBS. Differences in SARS-CoV pseudovirus entry between trypsin-treated and untreated cells are significant (*P*<0.5) for all IFITM-expressing cells. (**B**) In parallel, ACE2 cell-surface expression was measured on aliquots of Vero E6 cells used in (A). Vero E6 cells were labeled with Alexa 488-conjugated S-protein RBD of SARS-CoV and analyzed by flow cytometry. (**C**) IFITM protein expression in ACE2-expressing Vero E6 cells was measured by western blot with an anti-c-myc antibody (9E10), using aliquots of the same cells assayed in (A). Figs. (A)–(C) are representative of three experiments with similar results. (**D**) Cathepsin L activity in Vero E6 cells transduced with vector alone or stably expressing indicated IFITM proteins was measured fluorometrically and is shown as mean fluorescence. Cells treated with a cathepsin L inhibitor were used as a control. No statistically significant differences were observed between vector-transduced and IFITM-expressing cells. (**E**) Vero E6 cells transduced with vector alone or stably expressing the indicated IFITM proteins were labeled with MR-(FR)_2_ or with MR-(RR)_2_, which bind to the active forms of cathepsin L or B, respectively. Cells were labeled for 1 hour, fixed with formaldehyde, and analyzed by flow cytometry. In vector-transduced cells, mean fluorescence for MR-(FR)_2_ was 1175.7 and for MR-(RR)_2_ was 454.7. The modest enhancement for cathepsin B activity in IFITM2 and 3 expressing cells is significant (*P*<0.05), whereas no significant differences were observed in cathepsin L activity. Experiments in (D) and (E) are representative of two experiments with similar results.

Previous studies have shown that the cathepsin L dependence of SARS-CoV can be bypassed by addition of exogenous trypsin to ACE2-bound virions or pseudovirions, likely by inducing S-protein-mediated fusion at or near the plasma membrane [11], [16]. In contrast to cathepsin L-mediated fusion, exogenous trypsin promotes fusion at or near the plasma membrane. To localize IFITM-mediated restriction, we investigated the effect of trypsin treatment on SARS-CoV entry into IFITM-expressing cells. Vero E6 cells transduced to express ACE2 and IFITM1, 2 or 3, or with vector alone were incubated with SARS-CoV or MACV pseudoviruses. As expected, IFITM expression restricted SARS-CoV S protein-mediated entry, but not MACV GPC-mediated entry. In contrast, when SARS-CoV pseudovirions were bound to ACE2-expressing cells at 4°C and then incubated with trypsin at 37°C for a short time, entry into IFTIM-expressing cells was largely restored (Figs. 6A, B, and C). These data suggest that IFITM proteins restrict cathepsin-dependent entry of SARS-CoV in the lysosome, but cannot restrict trypsin-induced fusion at or near the plasma membrane. We did not observe in IFITM-expressing cells any decrease in cellular cathepsin activity that could readily account for this difference (Figs. 6D and E). These data show that IFITM-mediated restriction is not a consequence of a decrease in the activity of lysosomal cathepsins, and suggest that lysosomal pH, which activates cathepsins B and L, is similarly unaffected by IFITM protein expression.

### IFITM proteins do not interfere with virion access to acidic cellular compartments

**Figure 7:**
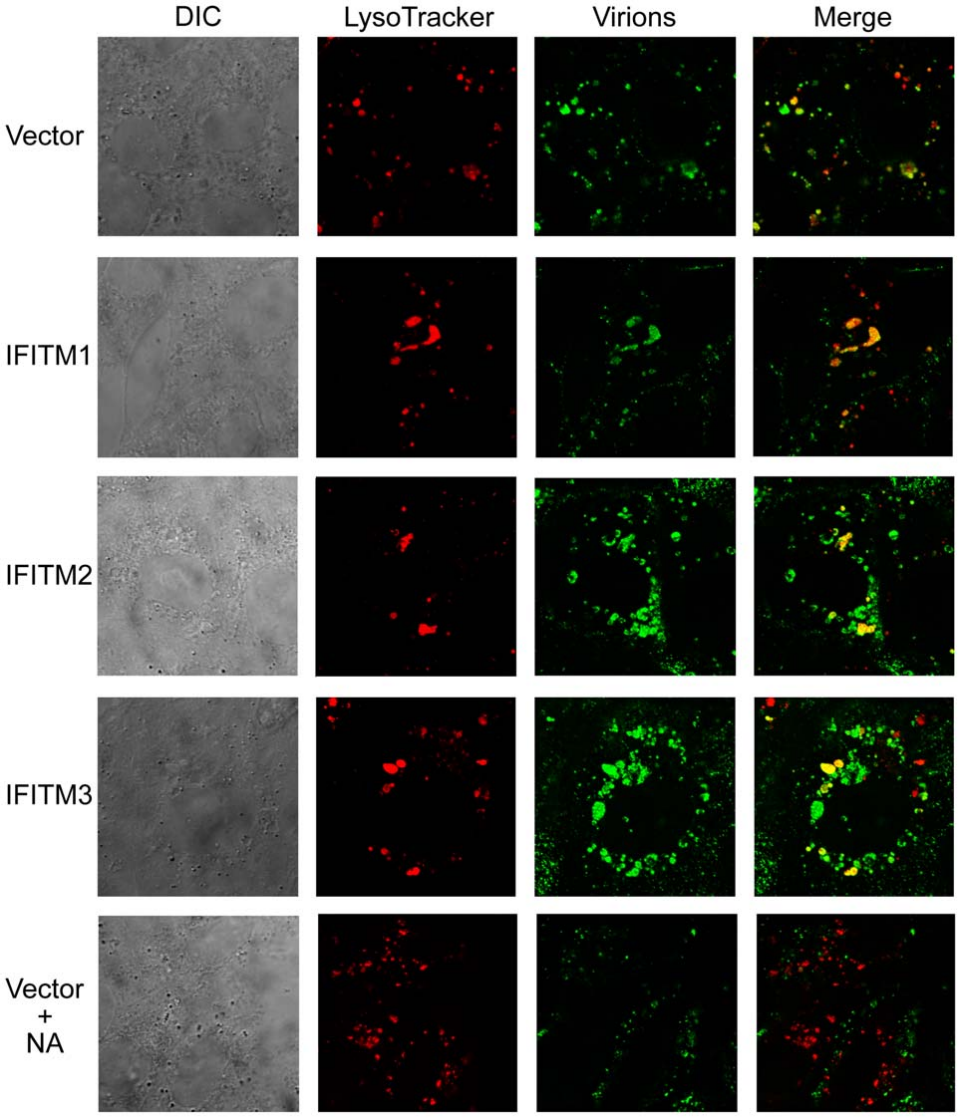
IFITM proteins do not interfere with virion access to acidic cellular compartments. Infectious influenza A/PR/8/34 (H1N1) virus was labeled with Alexa 488-conjugated murine anti-NA (N1) IgG2a (NA-112-S2.4) at 4°C for 16 hours (green). Vero E6 cells transduced to express the indicated IFITM proteins or with vector alone were spin-inoculated with labeled influenza A/PR/8/34 (H1N1) virus (M.O.I. = 100) at 4°C, washed twice with PBS, and incubated with medium containing 100 nM LysoTracker Red DND-99 (red), which labels low pH compartments, at 37°C for 100 minutes. Cells were then washed twice with PBS, fixed with formaldehyde, and imaged by confocal microscopy. Bottom panels show a control in which Vero E6 cells transduced with vector alone were pretreated with 1 U/ml bacterial neuraminidase (NA) for 24 hours before influenza A/PR/8/34 (H1N1) virus infection. Leftmost figures show differential interference contrast (DIC) and rightmost figures show the merged images of labeled virions and LysoTracker labeled cells.

The ability to circumvent IFITM-mediated restriction by bypassing the requirement for lysosomal cathespins raised the possibility that IFITM proteins interfere with access of virions to acidic cellular compartments. Using confocal microscopy, we monitored infectious IAV virions at 40, 70, and 100 minutes after association of virions with the plasma membrane in Vero E6 cells transduced to express IFITM1, 2, or 3, or with vector alone [22] (Figs. 7 and S4A). In all cases, labeled virions readily colocalized with cellular compartments labeled with LysoTracker, a fluorescent indicator of acidic compartments. In contrast, pretreatment of cells with bacterial neuraminidase, which removes the IAV receptor sialic acid from the cell surface (Fig. 7), or bafilomycin A_1_ (Fig. S4B), which prevents acidification of late endosomes or lysosomes, abolished colocalization of virions with acidic compartments. These data suggest that IFITM proteins do not interfere with the access of virions to low pH compartments necessary for fusion of IFITM-restricted viruses.

## Discussion

IFITM proteins play critical roles in the intrinsic and interferon-mediated control of IAV replication in human and murine cell lines [1]. In contrast to retroviral restriction factors – for example TRIM5α, APOBEC3G, or BST2/tetherin – they limit replication at a step mediated by the viral entry protein, likely before or during fusion of the viral and cellular membranes [23]. Although initially identified by an siRNA screen for factors that modulate IAV replication, they also inhibit an early step in the life-cycle of several flaviviruses including dengue and West Nile viruses [1].

The entry processes of IAV and flaviviruses both require low pH in an intracellular compartment to promote conformational changes in their entry proteins (HA and E protein, respectively) [6]. Here we investigate three viruses – two filoviruses and SARS-CoV – that similarly require access to acidic compartments to enter cells. However, unlike IAV and flaviviruses, activation of viral entry proteins of these viruses is not directly mediated by acidic pH; rather acidic pH is necessary to activate lysosomal proteases, which in turn cleave and activate these entry proteins. The MARV, EBOV, and SARS-CoV entry proteins each require cathepsin L activity to mediate fusion [10], [11], [12], [13]. In addition, EBOV requires cathepsin B activity. We observed that, like IAV and flaviviruses, the entry processes of all three cathepsin-dependent viruses are restricted by a range of human IFITM proteins and by their mouse and chicken orthologs.

It is not yet clear how IFITM proteins restrict these viruses, but this study considerably narrows the range of possible mechanisms. First, the number and diversity of restricted viruses tends to exclude mechanisms that might be unique to individual viruses. We previously showed that IFITM proteins do not decrease cell-surface expression of sialic acid, the primary attachment moiety for IAV [1]. Similarly, IFITM proteins do not significantly alter expression of the SARS-CoV receptor ACE2. Nor is it plausible that there is a physical interaction between the entry proteins of restricted viruses and the IFITM molecules, given the variation among these entry proteins and the ease with which such a mechanism might be evaded. However, all IFITM-restricted viruses described to date require access to late endosomal or lysosomal compartments. This observation raises the possibility that IFITM proteins interfere with endosomal trafficking or, alternatively, make these compartments unsuitable for fusion of restricted viruses.

Our data provide clear support for a mechanism that operates at a later stage in the endocytic pathway. Specifically, we circumvented restriction by complementing the cathepsin L requirement of SARS-CoV with exogenous trypsin, thereby promoting fusion at or near the plasma membrane. Moreover, we do not observe any IFITM-induced differences in the efficiency with which virions can access acidic compartments in the cell. Therefore, when viruses are restricted, they are restricted in a late endosomal or lysosomal compartment. We did not, however, observe a loss of cathepsin activity in IFITM-expressing cells, suggesting that this activity and the acidification that it requires are not grossly impaired by IFITM proteins. It is of course possible that these observations reflect underlying limitations in our ability to detect relevant differences. In particular, our ability to detect IAV in acidic compartments of IFITM expressing cells does not exclude the possibility that these compartments are distinct from those where productive infection can occur. Nonetheless, our data suggest that IFITM proteins inhibit viral entry by mediating a change in the properties of late endosomes or lysosomes. Two additional observations are consistent with this hypothesis. First, in some cell lines, enlarged vesicles could be observed in IFITM-expressing cells, although the scale of this enlargement did not correlate with the efficiency of viral restriction. Second, IFITM3 in particular colocalizes extensively with markers of late endosomes and lysosomes, but poorly with a marker of early endosomes (Fig. S5). Our ability to bypass IFITM restriction may also shed light on the course of SARS-CoV infection *in vivo*. In particular, following induction of an interferon response, replication of the virus is likely to be localized to tissues – for example the gastrointestinal and respiratory tracts – where trypsin-like proteases are abundant [16].

We also observed that different IFITM proteins restricted IAV and filoviruses with different efficiencies. In general, IFITM3 and its murine ortholog more efficiently restricted IAV than cathepsin-dependent viruses, as indicated by the marked increase in IAV entry, but not that of SARS-CoV, EBOV, or MARV, when endogenous IFITM3 was depleted in HeLa cells. In contrast, when endogenous IFITM1 was depleted in K562 cells, more efficient entry was observed for all IFITM-restricted viruses. Also, murine Ifitm5 and Ifitm6, and chicken Ifitm5 efficiently restricted entry mediated by both filovirus GP_1,2_ proteins, but had little or no effect on IAV HA-mediated entry. Thus the pattern of basal or interferon-induced expression of the IFITM proteins is likely to be an independent determinant of viral tropism and pathogenesis *in vivo*.

## Materials and Methods

### Cells

Human embryonic kidney 293T, human cervical carcinoma HeLa, and African green monkey epithelial Vero and Vero E6 cells were maintained in Dulbecco's minimal essential medium (DMEM; Invitrogen). Human lung epithelial A549 and human myelogenous leukemia K562 cells were grown in Roswell Park Memorial Institute (RPMI) 1640 medium (Invitrogen) and in Iscove's modified Dulbecco's medium (IMDM; Invitrogen), respectively. All media were supplemented with 10% fetal bovine serum (FBS; Invitrogen), 100 U/ml penicillin, and 100 µg/ml streptomycin (Invitrogen). A549 cells transduced with vector alone or stably expressing IFITM proteins were selected with 3 µg/ml puromycin (Invitrogen). Vero and Vero E6 cells transduced with vector alone or stably expressing IFITM proteins were maintained in growth medium supplemented with 4 µg/ml puromycin. HeLa or K562 cells stably expressing a control shRNA or shRNA targeting IFITM1, 2, 3, or 1/3 mRNA were also selected with 4 µg/ml puromycin. Human umbilical vein endothelial cells (HUVEC, Lonza) were maintained in endothelial cell growth medium-2 (EGM-2) supplemented with EGM-2 bulletkit (Lonza).

### Plasmids and constructs

A DNA segment encoding the c-myc tag sequence and the *Age*I restriction enzyme site was introduced into the pQCXIP vector (Clontech). Plasmids encoding various c-myc-tagged IFITM proteins were constructed by polymerase chain reaction (PCR) amplification of the coding regions of human IFITM1, 2, and 3, and mouse *Ifitm1*, *2*, *3*, *6*, and *7* genes using plasmids carrying respective cDNA (Open Biosystems) as templates [1]. PCR products were then digested and ligated into the *Age*I/*Bam*HI restriction sites of the pQCXIP vector. Codon-optimized chicken *Ifitm1*, and human, mouse, and chicken *Ifitm5* genes were synthesized (Genescript) and also cloned into the pQCXIP vector. pRS vector-based control shRNA and shRNA constructs targeting human IFITM1, 2, 3, or 1/3 mRNA were purchased from OriGene. The sequences of the control 29-mer and those targeting coding regions of IFITM1, 2, or 3 mRNA are as follows: IFITM1 – TGCACAAGGAGGAACATGAGGTGGCTGTG; IFITM2 – CCGCAGCGAGACCTCCGTGCCTGACCATG; IFITM3 –TCCTCATGACCATTCTGCTCATCGTCATC; IFITM1/3 – TGAATCACACTGTCCAAACCTTCTTCTCT; and control – GCACTACCAGAGCTAACTCAGATAGTACT.

### Pseudotyped murine leukemia viruses (MLVs) for transduction and infections assays

Plasmids and procedures used to generate pseudotyped MLV-GFP have been previously described, as have the various viral entry proteins used [18]. These entry proteins include influenza A virus HA proteins from A/PR/8/34 (H1N1) (H1(PR)), A/Udorn/72 (H3N2)(H3(Ud)), A/Thailand/2(SP-33)/2004(H5N1)(H5(Thai)), and A/FPV/Rostock/34 (H7N1)(H7(FPV)), glycoproteins (GP_1,2_) from Marburg virus Musoke (MARV) and Ebola virus Mayinga (EBOV), glycoprotein (GPC) from Machupo virus Carvallo (MACV), S protein from severe acute respiratory syndrome coronavirus Tor2 (SARS-CoV), and glycoprotein (GP) from amphotrophic MLV [18], [19], [20], [21].

To produce transducing viruses, 293T cells plated at 70% confluence in T75 culture flasks were transfected using the calcium phosphate method with 10 µg plasmid encoding MACV GPC, 15 µg plasmid DNA encoding MLV gag and pol, and 15 µg pQCXIP-based plasmids encoding various human, mouse, or chicken IFITM proteins or pRS-based plasmids encoding shRNA targeting human IFITM1, 2, 3, or 1/3 mRNA. 48 hours after transfection, culture supernatants were harvested and filtered through a 0.45 µM syringe filter (Nalgene).

Cells were then transduced by incubating transducing viruses mixed with 10 µg/ml of polybrene (Santa Cruz Biotechnology) and centrifuged at 4°C for 30 minutes at 4,000×g. Transduced cells were maintained in growth medium and employed for stable cell line selection or infection 48 hours later. The procedures for MLV-GFP pseudovirus infection were similar to that for transduction, except that spin inoculation was used and polybrene was not included. 48 hours after infection, infected cells were fixed with 1% formaldehyde (Polysciences) and analyzed by flow cytometry. To test the effect of type I interferon (IFN) on entry of MLV-GFP pseudotyped with various viral entry proteins, HeLa cells were treated with 5000 U/ml or 1000 U/ml human IFN-β (Antigenix America) for 48 hours before pseudovirus infection. Trypsin-bypass experiments were performed similarly, except that following spin inoculation at 4°C cells were incubated with 5 µg/ml trypsin or with PBS alone at 37°C for 13 minutes.

### Infectious filovirus replication assays

Propagation of MARV (Ci67 variant), EBOV (Kikwit variant), or EBOV-GFP (Mayinga variant) has been described [20], [24]. For filovirus infection, Vero E6 or A549 cells transduced to express IFITM proteins or with vector alone were incubated with MARV or with EBOV at a multiplicity of infection (MOI) of 1 or 15. The inocula were removed 1 h later and cells were washed 3 times with phosphate buffered saline (PBS). Culture supernatants were harvested in TRIzol (Invitrogen) 72 hours after infection, and virion yield was determined by quantitative reverse transcriptase-PCR (qRT-PCR). In brief, total RNA from culture supernatants of untreated cells (mock) or cells infected with MARV or EBOV was prepared using the MagMax 96 RNA Extraction Kit (Ambion). qRT-PCR assays were performed on an ABI PRISM 7900HT sequence detection system with the RNA UltraSenseTM one-step kit (Invitrogen) and TaqMan Probes (Applied Biosystems) according to manufacturers' instructions. The final concentrations used in the 20-µl reaction mix contained 5 µl of RNA, 0.4 µM of each primer, 0.2 µM probe, 4 µl of 5× reaction mix, 0.4 µl of Rox, and 1 µl of enzyme mix. The reaction was run as follows: reverse transcription at 50°C for 20 minutes; initial denaturation at 95°C for 2 minutes; amplification for 40 cycles at 95°C for 15 seconds, 60°C for 30 seconds. Serial 10-fold dilutions of the assayed (10^2^–10^7^ copies) virus were used as standards.

For imaging MARV infected cells, cells were fixed in 10%-buffered formalin (Val Tech Diagnostics) for 72 h and stained for high-content quantitative image-based analysis with a murine monoclonal antibody against MARV GP_1,2_ (9G4), followed by Alexa 488-conjugated goat anti-mouse IgG (Invitrogen). All infected cells were stained with Hoechst 33342 and HCS CellMask Red (Invitrogen). Images were acquired and analyzed on an Opera confocal reader (model 3842-Quadruple Excitation High Sensitivity (QEHS), Perkin Elmer), at two exposures using a 10× air objective.

### Infectious SARS-CoV replication assays

Propagation of SARS-CoV has been described [25]. Vero cells sorted to express moderate levels of ACE2 were transduced to express IFITM proteins or with vector alone were incubated with SARS-CoV (Urbani strain) at a multiplicity of infection (MOI) of 0.1 for 1 hour. 1, 6, 12, 18, 24, or 30 hours after infection, viral titer was determined by plaque assay [25].

### Immunofluorescence studies

ACE2 cell surface expression was measured by labeling cells with 20 µg/ml Alexa-488 or Alexa-649 (Pierce) conjugated S protein receptor-binding domain (RBD) of SARS-CoV for one hour [26]. Cells were then washed 3 times with PBS, fixed with 1% formaldehyde and analyzed by flow cytometry. To image pseudovirus infected cells, cells infected with various MLV-GFP pseudotypes were fixed with 4% formaldehyde for 20 minutes, permeablized with 0.2% Triton X-100 (Sigma) for 15 minutes, and counterstained with 25 ng/ml 4′,6-Diamidino-2-phenylindole (DAPI; Sigma) for 5 minutes. Images were taken using the Olympus IX51 fluorescence microscope and the DP controller software (objective 20×). Expression of human c-myc-tagged IFITM proteins in A549 cells was assayed using similar procedures. A549 cells transduced to express human IFITM proteins or with vector alone were fixed, permeablized, labeled with fluorescein isothiocyanate (FITC)-conjugated murine anti-c-myc IgG (9E10; Abcam) for one hour, and counterstained with DAPI. Cells were imaged using the same method.

Subcellular localization of human IFITM3 was measured by confocal microscopy. IFITM3-expressing A549 cells were washed twice with PBS and fixed in 50/50 acetone/methanol at −20°C for 10 minutes. Fixed cells were then washed with PBS twice, blocked with 10% goat serum in PBS for 30min, and labeled with various primary antibodies for 2 hours. Primary antibodies used in this study included murine anti-lysosomal-associated membrane protein 1 (LAMP1, H4A3, 1∶50 dilution; Santa Cruz Biotech) IgG1, murine anti-LAMP2 (H4B4, 1∶50 dilution; Santa Cruz Biotech) IgG1, murine anti-CD63 (FC-5.01, 1∶100 dilution; Invitrogen) IgG2a, rabbit anti-early endosomal antigen 1 (EEA1, 1∶100 dilution; Sigma) polyclonal antibody, and murine anti-c-myc antibodies (9E10, 1∶50 dilution, and 9E11, 1∶900 dilution; Santa Cruz Biotech). After primary antibody staining, cells were labeled with goat Alexa 488- or 568- conjugated secondary antibodies (1∶500 dilution; Invitrogen) for 1 hour and then analyzed by confocal microscopy using Leica TCS-SP1 laser confocal imaging system (objective 100×).

### Cathepsin activity assays

Cathepsin L activity was assayed using the cathepsin L activity kit following the protocol provided by the manufacturer (EMD). Cathepsin L activity of 2×10^5^ Vero E6 cells transduced to express IFITM proteins or with vector alone was assayed. A cathepsin B inhibitor (CA-074) was added in each tested sample to eliminate the interference from cathepsin B. To evaluate cathepsin B or L activities in live cells, Magic Red conjugated-(z-Leucine-Arginine)_2_ (MR-(RR)_2_), which binds to active cathepsin B, or MR conjugated- (z-Phenylalanine-Arginine)_2_ (MR-(FR)_2_), which binds to active cathepsin L, were used (Immunochemistry Technologies). Vero E6 cells transduced to express IFITM proteins or with empty vector were incubated with either MR-(RR)_2_ or MR-(FR)_2_ at 37°C. One hour later, cells were harvested, fixed with 1% formaldehyde and analyzed by flow cytometry.

### Western blots

Cells were lysed with either 1% Triton X-100 (Sigma) or 1% NP-40 (Thermo Scientific). Protein samples were prepared in reducing buffer and boiled for 5 minutes, analyzed by sodium dodecyl sulfate polyacrylamide gel electrophoresis (SDS/PAGE), and transferred to a polyvinylidene difluoride membrane (Invitrogen). Expression of various c-myc-tagged IFITM proteins was detected by 0.4 µg/ml murine monoclonal anti-c-myc antibody (9E10; Santa Cruz Biotechnology). Endogenous IFITM protein expression was probed by 0.4 µg/ml polyclonal rabbit anti-IFITM1 (FL-125, Santa Cruz Biotechnology), rabbit anti-IFITM2/3 (1∶2000 dilution; anti-IFITM2 antibody was from Proteintech Group which cross reacts to IFITM3 protein). β-actin recognized by 1 µg/ml murine monoclonal anti-β-actin antibody (Sigma) was used as a loading control.

### Virion trafficking assays

Influenza A virus A/PR/8/34 was purchased from American Type Culture Collection (ATCC) and was propagated in Madin-Darby canine kidney (MDCK) cells as described [18]. To label Influenza A/PR/8/34 virions, virions were incubated with 20 µg/ml Alexa 488 (Pierce)-conjugated monoclonal murine anti-NA (N1) IgG (NA-112-S2.4) at 4°C for 16 hours. (Antibody NA-112-S2.4 was generously provided by Dr. W. Gerhard, The Wistar Institute, US). Virions were used for infection assays directly after labeling.

To monitor virion trafficking, Vero E6 cells transduced to express IFITM proteins or with vector alone were incubated with labeled influenza A/PR/8/34 virions at an MOI of 10 or 100 and centrifuged at 4°C for 30 minutes at 4000×g. Cells were then washed three times with PBS and maintained in medium containing 100 nM LysoTracker Red DND-99 (Invitrogen) at 37°C. 40, 70, or 100 minutes later, cells were fixed with 4% formaldehyde and imaged with a Leica DMRBE Microscope and Leica TCS-SP1 laser confocal imaging system (objective 100×). Control cells were pre-treated with 1 U/ml neuraminidase from *Clostridium perfringens* (Sigma) for 24 hours or with 100 nM bafilomycin A_1_ (Baf A1, Sigma) for 6 hours before influenza A virus infection.

## Supporting Information

Figure S1 Viral infection and endogenous IFITM expression in cells assayed. **(A)** A549 cells transduced to express indicated IFITM proteins or with vector alone were infected with MLV-GFP pseudotyped with MARV or EBOV glycoprotein as indicated (green). Two days later, cells were fixed with formaldehyde, permeablized by Triton X-100, and counterstained with DAPI (blue). Images were taken using fluorescence microscopy. **(B)** 293T, A549, or Vero E6 cells were treated with 5000 U/ml IFN-β, 250 ng/ml IFN-γ, or with medium alone, as indicated, for 48 hours. Expression of IFITM proteins was assayed by western blot using the indicated anti-IFITM1 or anti-IFITM2/3 antibody. (C) Vero E6 cells transduced to express indicated IFITM proteins or with vector alone were incubated with live MARV. 72 hours later cells were fixed with formaldehyde for 72 hours, labeled with murine monoclonal antibody against MARV GP_1,2_ (9G4), counterstained with Hoechst 33342, and imaged by confocal microscopy.
(3.61 MB TIF)

Figure S2 Pseudovirus entry, ACE2 and IFITM expression. **(A)** Experiment similar to that in Fig. S1A except that control or IFITM3 shRNA-expressing HeLa cells were infected with the indicated pseudoviruses. **(B)** ACE2 expression in cells used in Fig. 4D was assayed using Alexa 649-conjugated S protein RBD of SARS-CoV, analyzed by flow cytometry, and shown as mean fluorescence intensity. **(C)** ACE2 expression in cells used in Fig. 4G was assayed by the same method described in (B). **(D)** A549 cells transduced to express c-myc-tagged human IFITM2, IFITM5, or with vector alone were fixed, permeablized, and stained with FITC-conjugated anti-c-myc antibody (green). Cells were then counterstained with DAPI (blue) and imaged using fluorescence microscopy.
(3.19 MB TIF)

Figure S3 Alignment of IFITM orthologs used in this study. **(A)** A representation of the topology of the IFITM proteins embedded in a cellular membrane. **(B)** An alignment of human, mouse, and chicken IFITM orthologs, with topological features indicated above. Red indicates conserved residues.
(1.01 MB TIF)

Figure S4 IFITM proteins do not interfere with virion access to acidic cellular compartments. **(A)** Experiment similar to that in Fig. 7, except that Vero E6 cells transduced to express IFITM3 or with vector alone were infected with labeled influenza A/PR/8/34 virus at a MOI of 10. Infected cells were washed twice with PBS, fixed with formaldehyde 40, 70, or 100 minutes as indicated after incubation with labeled viruses. Leftmost figures show DIC images and rightmost figures show merged images of labeled virions and LysoTracker labeled cells. **(B)** Two different controls for (A) are shown. Vero E6 cells transduced with vector alone were pretreated with 100 nM bafilomycin A_1_ (BafA1) for 6 hours or with 1 U/ml bacterial neuraminidase (NA) for 24 hours before incubation with influenza A/PR/8/34 (H1N1) virus. Images were taken by confocal microscopy 100 minutes later.
(8.16 MB TIF)

Figure S5 IFITM3 colocalizes to late endosomal lysosomal markers. A549 cells expressing c-myc-tagged IFITM3 were fixed with formaldehyde, permeablized, and labeled with the anti-c-myc antibody and antibodies against indicated organelle markers. Cells were then imaged by confocal microscopy. Leftmost figures show DIC images and rightmost figures show merged images of IFITM3 (red) and the indicated organelle markers (green).
(3.57 MB TIF)
