# Supplementary figures and images for: Distinct Patterns of IFITM-Mediated Restriction of Filoviruses, SARS Coronavirus, and Influenza A Virus
Source: PLoS Pathog. 2011 Jan 6;7(1):e1001258. doi: 10.1371/journal.ppat.1001258 (PMC3017121; doi:10.1371/journal.ppat.1001258)

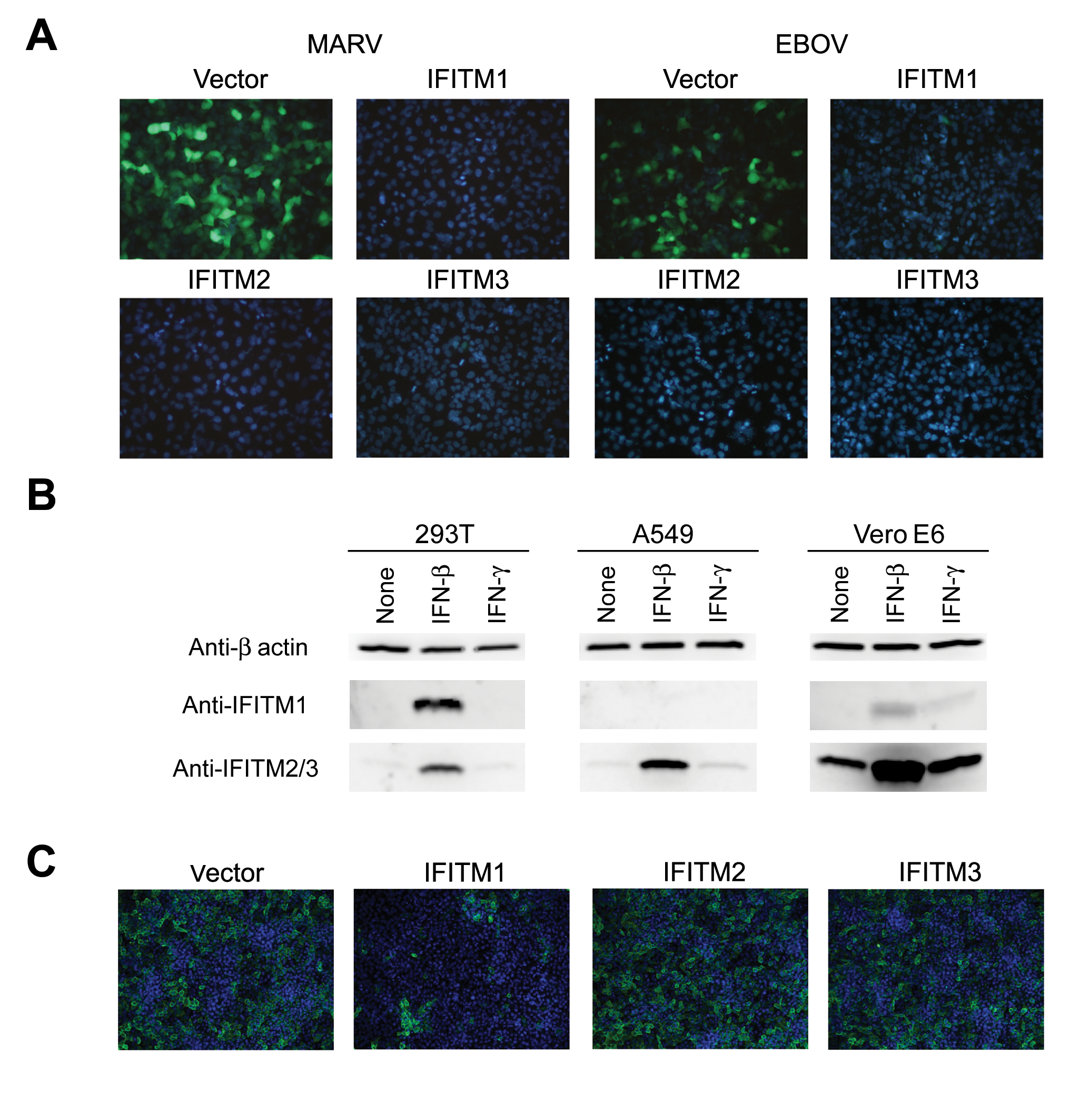

Supplement: Figure S1 — Viral infection and endogenous IFITM expression in cells assayed. (A) A549 cells transduced to express indicated IFITM proteins or with vector alone were infected with MLV-GFP pseudotyped with MARV or EBOV glycoprotein as indicated (green). Two days later, cells were fixed with formaldehyde, permeablized by Triton X-100, and counterstained with DAPI (blue). Images were taken using fluorescence microscopy. (B) 293T, A549, or Vero E6 cells were treated with 5000 U/ml IFN-β, 250 ng/ml IFN-γ, or with medium alone, as indicated, for 48 hours. Expression of IFITM proteins was assayed by western blot using the indicated anti-IFITM1 or anti-IFITM2/3 antibody. (C) Vero E6 cells transduced to express indicated IFITM proteins or with vector alone were incubated with live MARV. 72 hours later cells were fixed with formaldehyde for 72 hours, labeled with murine monoclonal antibody against MARV GP1,2 (9G4), counterstained with Hoechst 33342, and imaged by confocal microscopy. (3.61 MB TIF) [file ppat.1001258.s001.tif]

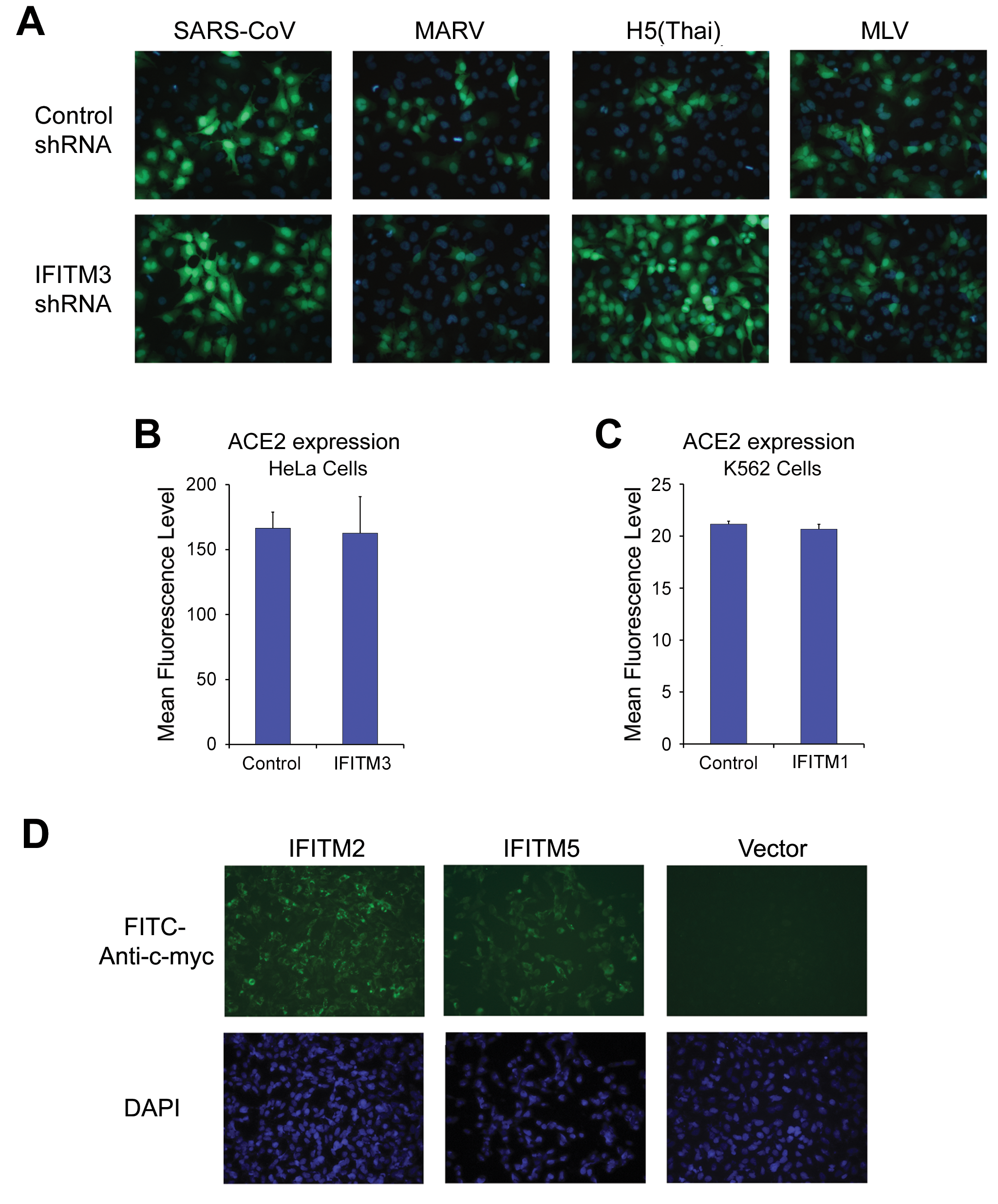

Supplement: Figure S2 — Pseudovirus entry, ACE2 and IFITM expression. (A) Experiment similar to that in Fig. S1A except that control or IFITM3 shRNA-expressing HeLa cells were infected with the indicated pseudoviruses. (B) ACE2 expression in cells used in Fig. 4D was assayed using Alexa 649-conjugated S protein RBD of SARS-CoV, analyzed by flow cytometry, and shown as mean fluorescence intensity. (C) ACE2 expression in cells used in Fig. 4G was assayed by the same method described in (B). (D) A549 cells transduced to express c-myc-tagged human IFITM2, IFITM5, or with vector alone were fixed, permeablized, and stained with FITC-conjugated anti-c-myc antibody (green). Cells were then counterstained with DAPI (blue) and imaged using fluorescence microscopy. (3.19 MB TIF) [file ppat.1001258.s002.tif]

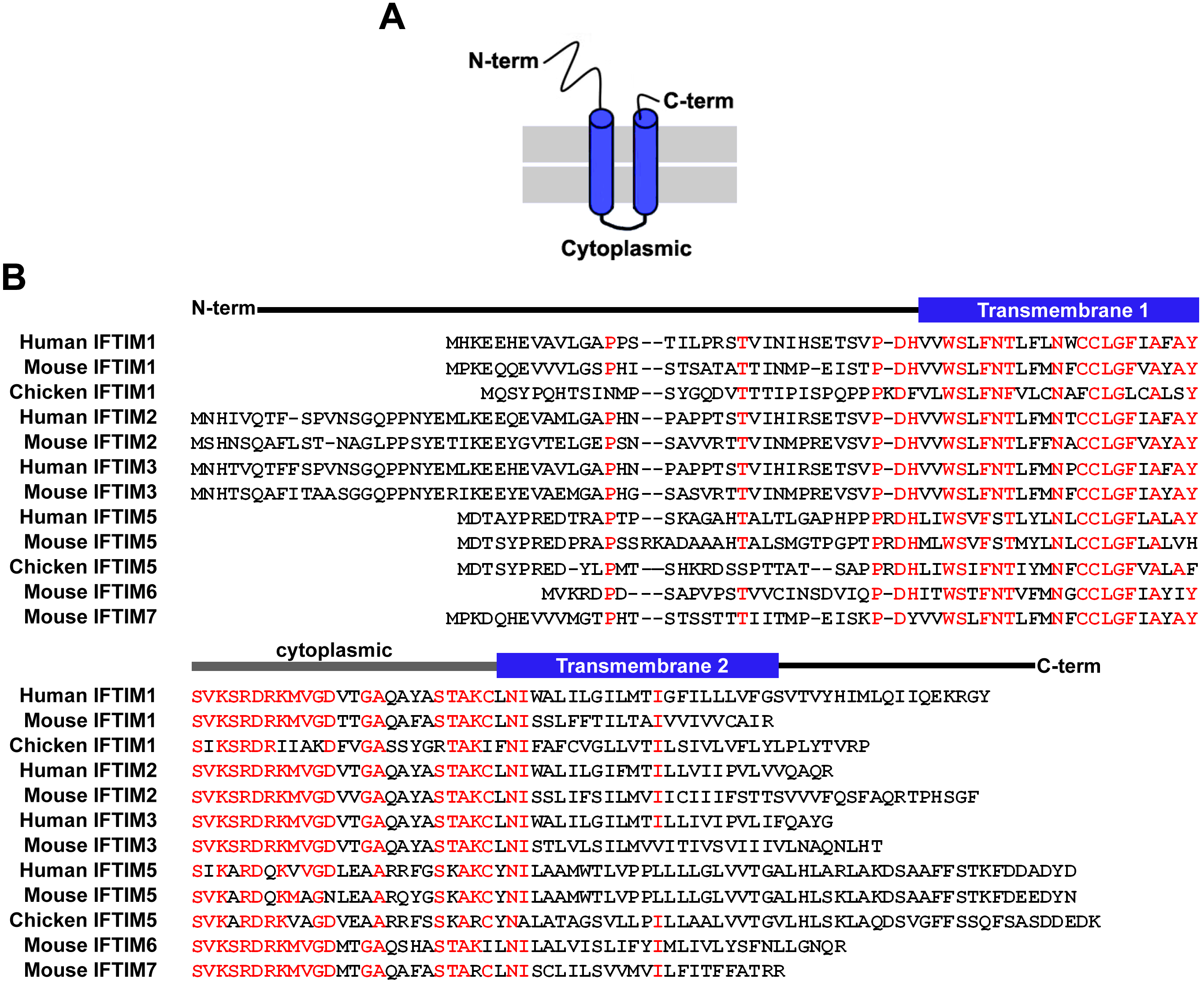

Supplement: Figure S3 — Alignment of IFITM orthologs used in this study. (A) A representation of the topology of the IFITM proteins embedded in a cellular membrane. (B) An alignment of human, mouse, and chicken IFITM orthologs, with topological features indicated above. Red indicates conserved residues. (1.01 MB TIF) [file ppat.1001258.s003.tif]

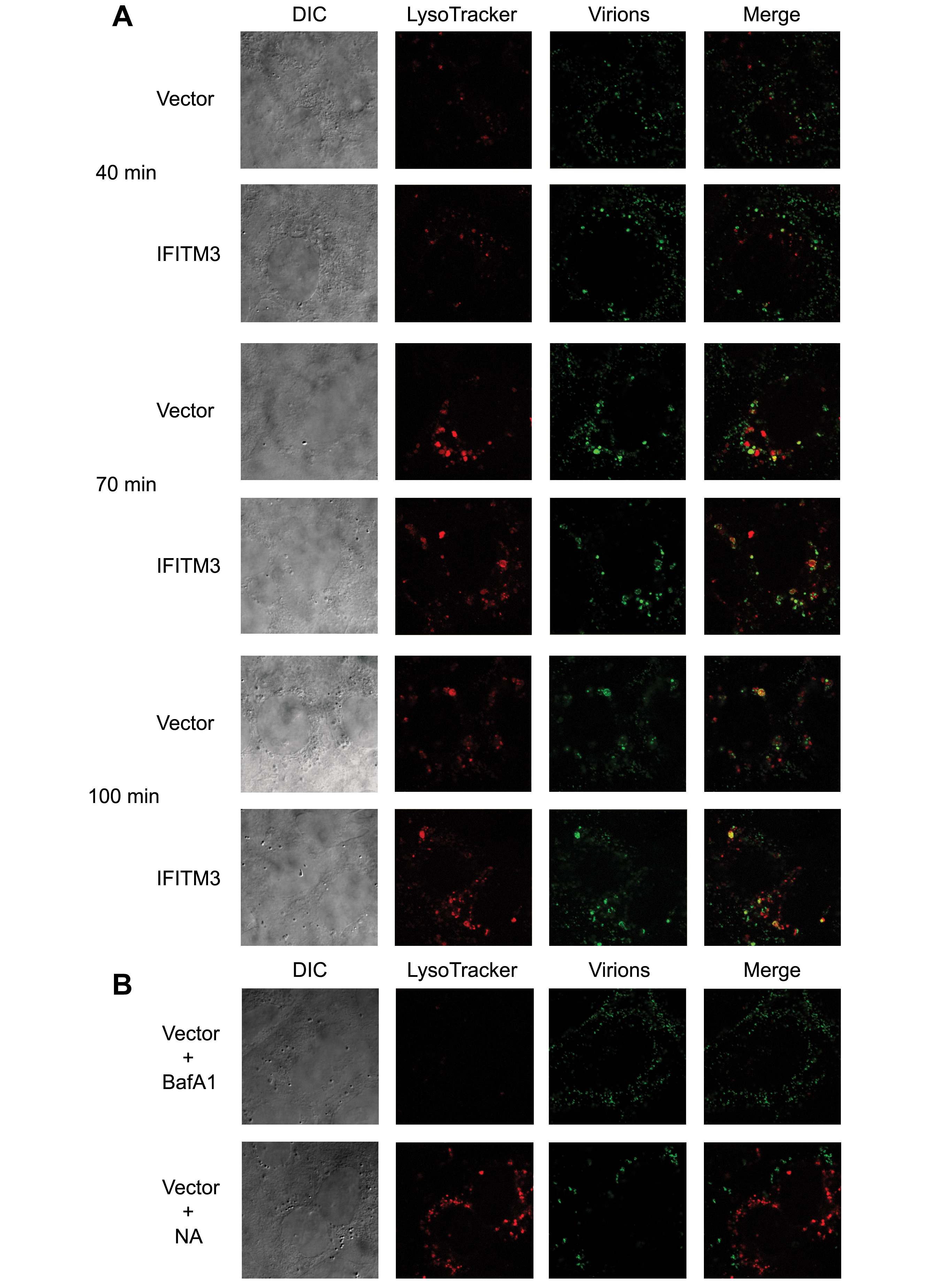

Supplement: Figure S4 — IFITM proteins do not interfere with virion access to acidic cellular compartments. (A) Experiment similar to that in Fig. 7, except that Vero E6 cells transduced to express IFITM3 or with vector alone were infected with labeled influenza A/PR/8/34 virus at a MOI of 10. Infected cells were washed twice with PBS, fixed with formaldehyde 40, 70, or 100 minutes as indicated after incubation with labeled viruses. Leftmost figures show DIC images and rightmost figures show merged images of labeled virions and LysoTracker labeled cells. (B) Two different controls for (A) are shown. Vero E6 cells transduced with vector alone were pretreated with 100 nM bafilomycin A1 (BafA1) for 6 hours or with 1 U/ml bacterial neuraminidase (NA) for 24 hours before incubation with influenza A/PR/8/34 (H1N1) virus. Images were taken by confocal microscopy 100 minutes later. (8.16 MB TIF) [file ppat.1001258.s004.tif]

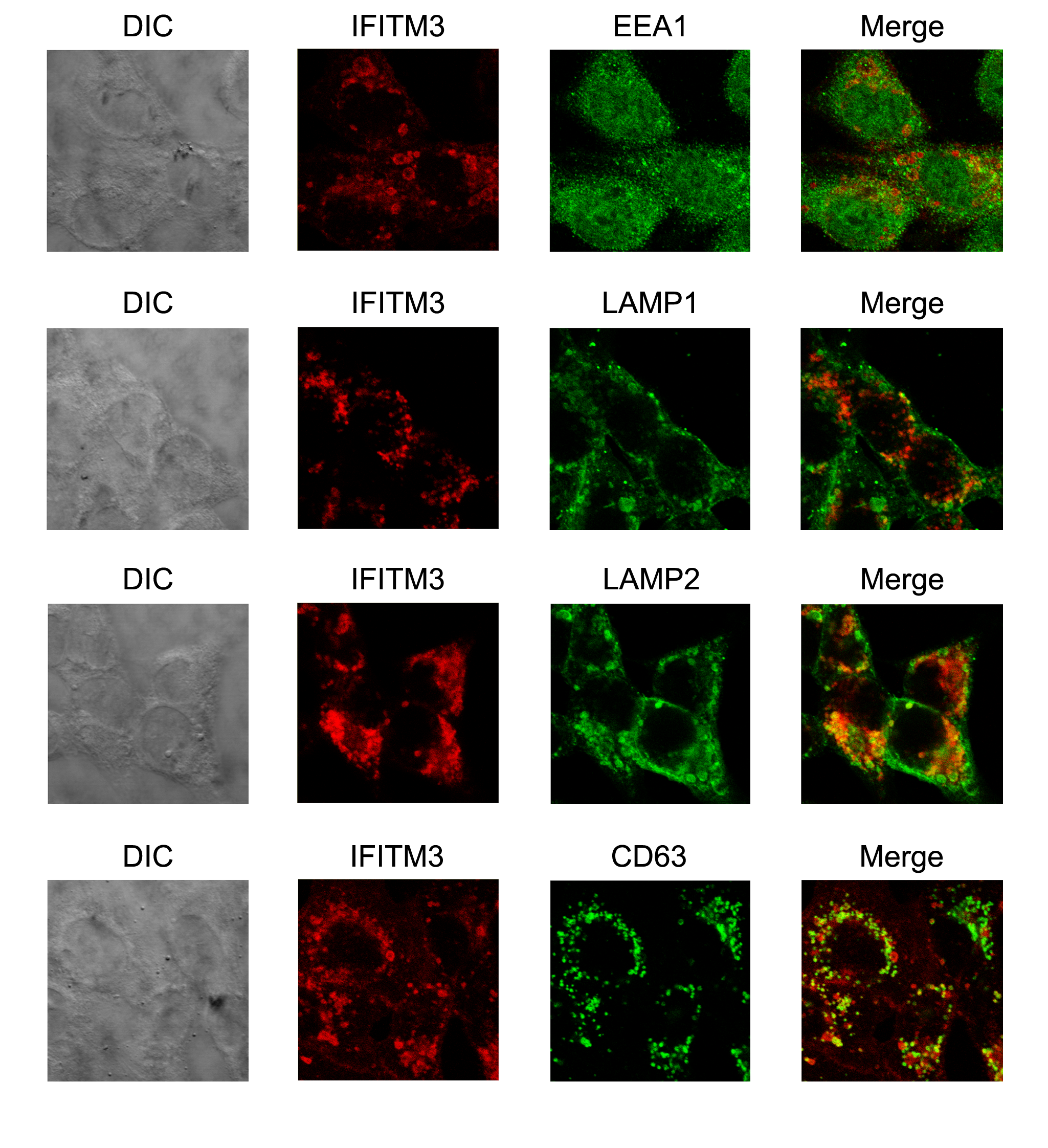

Supplement: Figure S5 — IFITM3 colocalizes to late endosomal lysosomal markers. A549 cells expressing c-myc-tagged IFITM3 were fixed with formaldehyde, permeablized, and labeled with the anti-c-myc antibody and antibodies against indicated organelle markers. Cells were then imaged by confocal microscopy. Leftmost figures show DIC images and rightmost figures show merged images of IFITM3 (red) and the indicated organelle markers (green). (3.57 MB TIF) [file ppat.1001258.s005.tif]
